# Three-dimensional flow structures past a bio-prosthetic valve in an *in-vitro* model of the aortic root

**DOI:** 10.1371/journal.pone.0194384

**Published:** 2018-03-16

**Authors:** David Hasler, Dominik Obrist

**Affiliations:** ARTORG Center for Biomedical Engineering Research, University of Bern, Bern, Switzerland; Texas A&M University System, UNITED STATES

## Abstract

The flow field past a prosthetic aortic valve comprises many details that indicate whether the prosthesis is functioning well or not. It is, however, not yet fully understood how an optimal flow scenario would look, i.e. which subtleties of the fluid dynamics in place are essential regarding the durability and compatibility of a prosthetic valve. In this study, we measured and analyzed the 3D flow field in the vicinity of a bio-prosthetic heart valve in function of the aortic root size. The measurements were conducted within aortic root phantoms of different size, mounted in a custom-built hydraulic setup, which mimicked physiological flow conditions in the aorta. Tomographic particle image velocimetry was used to measure the 3D instantaneous velocity field at various instances. Several 3D fields (e.g. instantaneous and mean velocity, 3D shear rate) were analyzed and compared focusing on the impact of the aortic root size, but also in order to gain general insight in the 3D flow structure past the bio-prosthetic valve. We found that the diameter of the aortic jet relative to the diameter of the ascending aorta is the most important parameter in determining the characteristics of the flow. A large aortic cross-section, relative to the cross-section of the aortic jet, was associated with higher levels of turbulence intensity and higher retrograde flow in the ascending aorta.

## Introduction

The flow field in the ascending aorta (AAo) and in the sinus of Valsalva (SOV) is a footprint of the aortic valve (AV) prosthesis after aortic valve replacement. It contains information about the valve performance at several levels: Strong gradients in the AAo velocity field cause high shear stresses possibly leading to blood damage [1, 2]. Turbulent flow in the AAo is associated with increased energy losses, and impingement of the jet-like outflow from the AV orifice may lead to endothelial damage [3–5]. Zones of low flow velocities increase blood residence time in the aortic root, promoting the risk of thrombus formation [6, 7]. Finally, structure and magnitude of the flow in the SOV is directly related to the valve leaflet kinematics, which is a determinant for acute valve performance and thrombogenicity [8]. Given the wide range of different prosthetic designs, implantation positions and different aortic root dimensions, a great variety of distinct flow scenarios is possible. A better understanding of the determinants for particular flow scenarios is needed because they are strongly connected to clinical concerns after AV replacement such as early valve thrombosis and structural valve deterioration [9, 10].

First efforts at describing the aortic valve fluid mechanics in detail date back to the late 1960’s: Bellhouse and Talbot (1969) and Bellhouse (1969) investigated the heart valve mechanics and the surrounding flow [11, 12]. Of special concern was the SOV and its role in valve closing. They presented a dynamical theory for the so-called aortic sinus vortex (ASV), which establishes in the SOV during systole. The ASV supposedly governs the pressure distribution on the aortic side of the valve leaflet such that valve closure is initiated before the flow direction reverses. Van Steenhoven and Van Dongen (1979) contradicted this theory and showed that (early) valve closure is mainly due to the flow deceleration between the leaflets and that it also works for stagnant flow conditions in the SOV [13]. Peskin (1982) put these controversial views into broader context, making clear that whether the ASV exists and how the valve closure is initiated are two separate questions [14].

The first clear evidence for the existence of ASVs was reported by Kilner *et al*. (1993) based on *in-vivo* results from 3D magnetic resonance velocity mapping in a healthy patient [15]. Using similar methods, 3D *in-vivo* flow patterns in the aortic sinus and in the AAo were further investigated in a number of studies [16, 17]. Although yielding further evidence for the existence of ASV, significant differences in vortex strength and incidence were found between the three sinus portions (coronary and non-coronary sinuses) and between different patients. Given the wide range of aortic root geometries and prosthetic valve designs, many situations are possible with unfavorable flow conditions in the SOV, e.g., regions of stagnant flow [6, 7]. In any case, an ASV can only be established if momentum is transported into the SOV. Such a momentum transport is established, for example, if the flow through the AV impinges on the aortic wall at or even below the sino-tubular junction (STJ) such that part of the flow is redirected into the SOV. This has already been pointed out by Bellhouse and Talbot (1969) who reported that the flow through the valve had a point of dividing streamlines at the STJ [11]. Momentum transport into the SOV was well established in most other experimental studies of ASV [13, 18, 19]. However, effective momentum transport is not guaranteed per se because the flow impingement described above depends on the size and directionality of the aortic jet (AJ) and on the particular aortic root geometry. For instance, larger STJ diameters may lead to a flow configuration where the AJ issuing from the valve orifice is too far away from the SOV, such that no momentum transport is established. This may be the case for patients with aortic stenosis, which has been connected to a significant widening of the aortic root [20, 21]. Therefore, it is not possible to identify a unique typical geometry and flow configuration and we will show in the following that the classical ASV according to Bellhouse does not exist for some aortic root geometries.

Furthermore, it is to be expected that the intensity and character of turbulent flow developing past AV prostheses is significantly affected by aortic root geometry and AV design [22]. However, quantitative studies on turbulent flow due to AV prostheses (e.g. [23, 24]) remain quite sparse. A better understanding this effect will allow for better prosthesis design and for improved AV prosthesis selection in function of the patient-specific aortic root geometry.

In the present *in-vitro* study, we studied the three-dimensional character of flow in the vicinity of a bio-prosthetic AV by tomographic particle imaging velocimetry (TOMO PIV). By analyzing the 3D flow field, we aimed at a more detailed understanding of the fluid dynamics in function of the aortic root size. We investigated two main questions of relevance for clinical outcomes: What is the effect of aortic root size on turbulence intensity and shear rates in the AAo? What is the effect of aortic root size on the flow (topology and magnitude) in the SOV?

## Materials and methods

**Fig 1:**
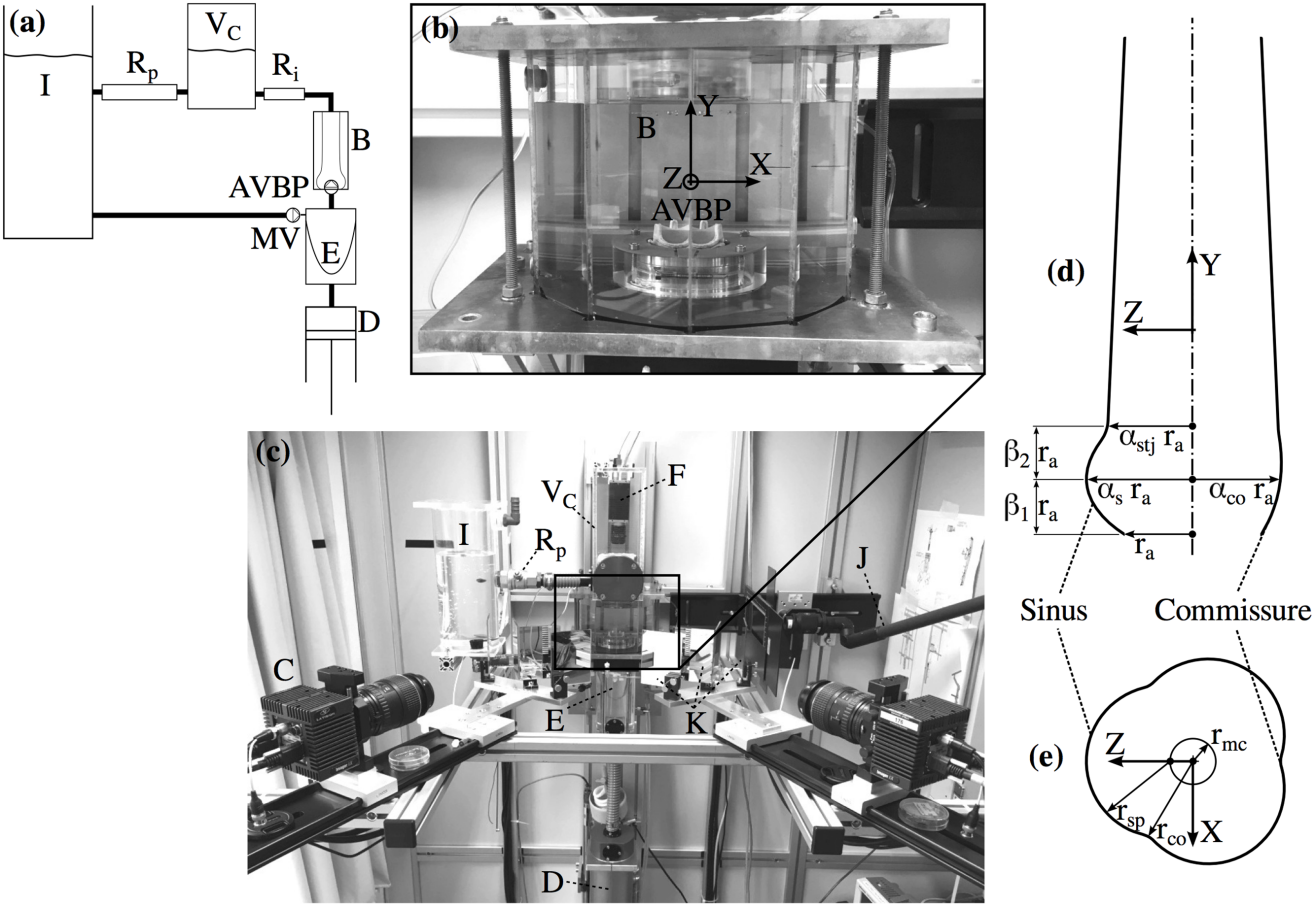
Experimental setup and aortic root scheme. Schematic of the flow loop (a). Test cell (b) containing the AVBP and the silicone phantom (B, immersed in blood analog fluid). Experimental setup (c) showing the CCD Cameras for TOMO PIV (C) and POA measurements (F); the piston pump (D) controlling the contraction and expansion of the silicone ventricle (E); elements of the flow loop: the compliance chamber with air volume *V*_C_, the peripheral resistance *R*_p_, and the tank (I); a light guide for volume illumination with a Nd:YAG laser (J); and the mirror setup for view-doubling (K). Parametrization of the aortic root (d) in axial plane at *X* = 0. Parametrization of SOV (e) in a cross-section at *β*_1_*r*_a_ above the annulus.

The experimental setup (Fig 1) consisted of the following main components: a pulsatile flow loop (pulse duplicator) driven by a piston pump that was tuned to replicate physiological flow conditions; a test cell with the AVBP in a transparent silicone phantom of the aortic root; two pressure transducers upstream (left ventricular position) and downstream (aortic position) of the AVBP; two 8M CCD cameras (LaVision GmbH, Göttingen, Germany) together with a mirror setup to acquire images for TOMO PIV from four different viewing angles. The cameras were mounted in a linear configuration with a total aperture angle of 90° and an angle of 30° between each camera view. A third camera (piA640-210gc, Basler AG, Ahrensburg, Germany) was mounted in axial position to record images of the valve orifice. More details on the technical protocol for the TOMO PIV measurements, e.g. calibration procedure and image post-processing, are reported in earlier work by the authors [25].

The flow loop was filled with blood analog fluid that flowed from an open tank into a silicone ventricle, passing a mechanical valve in mitral position. The silicone ventricle was mounted in a sealed chamber that was filled with water and connected to the piston pump controlling expansion and compression of the silicone ventricle. During compression of the ventricle, the blood analog fluid was pumped through an outflow tract, through the AVBP and through the thick-walled aortic root phantom. Further downstream the flow loop comprised an intermediate resistance *R*_i_ and a chamber with an enclosed air volume *V*_C_ mimicking arterial compliance. Before flowing back into a tank, the fluid passed a second resistance *R*_p_ modeling peripheral resistance of the systemic circulation. The parameters of the flow loop (*V*_C_, *R*_i_, *R*_p_) were first estimated using a lumped parameter model and then fine-tuned during operation of the setup.

### Aortic root model

Four different silicone phantoms of the aortic root were manufactured using ELASTOSIL RT 601 (Wacker Chemie AG, Munich, Germany). They were casted in a mold using 3D-printed cores in the shape of the aortic root lumen. A simplified, straight aortic root geometry was developed and scaled to obtain different dimensions for each phantom. The design of the aortic root (see Fig 1d and 1e) was defined by the diameter *d*_a_ = 2*r*_a_ of the aortic annulus and the following scaling parameters: the SOV parameter *α*_s_, the commissures parameter *α*_co_, the STJ parameter *α*_stj_, and the SOV height parameters *β*_1_ and *β*_2_.

Three aortic root phantoms had medium (M), large (L) and small (S) size. In the fourth phantom (NS) the SOV was omitted completely. The design of the medium phantom (M) was in accordance with anatomical data reported in [20, 21], valid for cases of severe aortic stenosis. In S and L, all dimensions (except for the aortic annulus radius) were scaled by ±10% with respect to M. This corresponds to the standard deviations of the dimensions reported in [21] and threfore the different phantoms represent typical inter-patient variations. Hence, S and L comprised roughly 30% less, respectively more, volume compared to M. The size of the NS phantom was the same as M, but without SOV. Instead, the circular lumen increased smoothly from the annulus toward the STJ. Table 1 lists the geometric parameters for each phantom.

**Table 1:** Parameter values for the aortic root phantom geometry.

Aortic Root	*α*_s_	*α*_co_	*α*_stj_	*β*_1_	*β*_2_
NS	-	-	1.25	0.70	0.80
S	1.40	1.13	1.13	0.63	0.72
M	1.55	1.25	1.25	0.70	0.80
L	1.70	1.38	1.38	0.77	0.88

The annulus radius was *r*_a_ = 11 mm for all geometries. The height and the radius of the SOV were (*β*_1_ + *β*_2_)*r*_a_ and *r*_s_ = *α*_s_*r*_a_, respectively, and the radius at the sino-tubular junction was *α*_stj_*r*_a_. Further, the distance of the maximum cross-section of the SOV was chosen *β*_1_*r*_a_ away from the annulus, and its shape was defined by the SOV radius *r*_sp_, the radius of the mid-circle *r*_mc_ and the commissure radius *r*_co_ = *α*_co_*r*_a_. Using the geometric constraints *r*_sp_ + *r*_mc_ = *α*_s_*r*_a_ and $r_{\text{sp}}^{2}=r_{\text{co}}^{2}+r_{\text{mc}}^{2}-r_{\text{co}}r_{\text{mc}}$ (law of cosines for an angle of *π*/6 between the commissure and the *Z*-axis), the following relations for *r*_mc_ and *r*_sp_ were derived:

$$\begin{array}{c} r_{\text{mc}}=(\frac{\alpha_{\text{co}}^{2}-\alpha_{s}^{2}}{\alpha_{\text{co}}-2\alpha_{s}})r_{a}\text{,} \end{array}$$ (1)

$$\begin{array}{c} r_{\text{sp}}=(\frac{\alpha_{\text{co}}\alpha_{s}-\alpha_{s}^{2}-\alpha_{\text{co}}^{2}}{\alpha_{\text{co}}-2\alpha_{s}})r_{a}\text{.} \end{array}$$ (2)

The phantoms were designed as thick-walled, nearly rigid models of the aortic root. They showed approximately 1% deformation during the experiments which is less than the physiological aortic deformation during a heartbeat [26].

For all measurements we used an *Edwards Intuity Elite* sutureless AVBP (Edwards Lifesciences, Irvine, CA, United States) with leaflets made from bovine pericard and with a nominal diameter of 21 mm. It was mounted on a socket at the bottom of the test cell reaching inside the aortic root phantom. The valve height with respect to the annulus was 14.5 mm. This resulted in axial distances between the trailing edge of the valve leaflets in open position and the STJ in axial direction of 2.0 (M), 0.4 (S), 3.7 (L) and 2.0 mm (NS).

### Blood analog fluid

The refraction index of the blood analog fluid was matched to that of the silicone phantom to minimize optical distortions during image acquisition. In addition, the kinematic viscosity of the fluid was close to that of blood. To meet both criteria, we used a water—glycerol (Dr. Grogg Chemie AG, Deisswil, Switzerland)—sodium chloride (Sigma-Aldrich Corporation, St. Louise, MO, USA) solution with fractions of 0.494, 0.340, 0.166 by weight, respectively. The density of the fluid was *ρ* = 1200 kg/m^3^ and its kinematic viscosity was *ν* = 4.7 mm^2^/s at a temperature of 22 °*C*.

The fluid was seeded with fluorescent PMMA microparticles (density *ρ*_p_ = 1180 kg/m^3^) with Rhodamin B coating (Microparticles GmbH, Berlin, Germany) and a diameter of *d*_p_ = 30 – 50 *μ*m. Assuming a reference velocity of *U* = 2 m/s, the Stokes number for these particles was St = *τU*/*d*_p_ ≈ 0.8 with a particle relaxation time of *τ* = *ρ*_p_*d*_p_/(18*ρν*) ≈ 21 *μ*s [27].

### Projected orifice area

Planimetric measurements were performed to determine the projected orifice area (POA) of the AVBP. To this end, images of the AVBP were recorded in an axial view at 200 frames per second during five subsequent pulses. Back-illumination allowed segmenting of the pixels in the orifice area using a threshold intensity value. Finally, the POA was quantified after an affine transform of each recorded image based on three well-defined landmarks (AVBP struts) to reduce perspective image distortion.

### TOMO PIV

The measurement volume for TOMO PIV comprised the lumen of the aortic root phantom extending from the top of the AVBP to two annulus diameters downstream of the STJ. In addition, separate TOMO PIV measurements were conducted in the front-facing SOV (aligned with positive *Z*-axis). We used the commercial TOMO PIV package DaVis 8.3 (LaVision GmbH, Göttingen, Germany) to analyze the raw camera images. The resulting instantaneous flow fields, **U**(**X**, *t*) = [*U*_*x*_(**X**, *t*), *U*_*y*_(**X**, *t*), *U*_*z*_(**X**, *t*)] with **X** = [*X*, *Y*, *Z*], comprised approximately 400′000 (AAo flow domain), respectively 20′000 (SOV flow domain), uniformly distributed 3D velocity vectors. Further details on the TOMO PIV measurements are given in [25] and in S1 Appendix.

### Experimental protocol

All experiments were performed with a pulsatile flow at 72 beats per minute (period *T* = 60/72 s) with a maximum flow rate of *Q*_max_ = 20 l/min and a systolic-diastolic ratio of 1/3 to 2/3. The resulting stroke volume was 68 ml corresponding to a cardiac output of 4.9 l/min. The relevant Reynolds and Womersley numbers were

$$\begin{array}{c} \text{Re}=\frac{4Q_{\text{max}}}{d_{\text{jet}}\pi \nu }\approx 6^{\prime }000\;\text{and}\;\alpha =r_{a}\sqrt{\frac{2\pi }{T\nu }}\approx 15\text{,} \end{array}$$ (3)

where *d*_jet_ ≈ 15 mm is a typical AJ diameter estimated from the POA of the M phantom. At this Reynolds number, flow instabilities were expected.

TOMO PIV measurements were triggered with respect to the pump motion. The simultaneous pressure and POA measurements were triggered with the same signal provided by the pump control unit. Measurements past the valve and in the SOVs were done sequentially because they required different pulse delays.

We performed phase-locked measurements at *t*_*j*_ = *j*d*t*, with *j* = 0, 1, …, 12 and d*t* = 0.03 s covering the whole systole as well as early diastole from *t* = 0 to 0.36 s. From every double-frame recording, the instantaneous velocity field **U**(**X**, *t*_*j*_) was calculated. From *N* = 16 separate instantaneous velocity fields for each *t*_*j*_, we computed the flow statistics.

Because the mean flow velocities were nearly zero at the end of diastole, we considered the flow as statistically periodic. Therefore, mean values at *t*_*j*_ were computed by phase-averaging, 〈⋅〉_*j*_. For the velocity field **U**, this resulted in

$$\begin{array}{c} {\left\langle U\right\rangle }_{j}=\frac{1}{N}\sum_{k=1}^{N}\;U\left(X,t_{j}+kT\right)\text{.} \end{array}$$ (4)

We used the Reynolds decomposition **U** = 〈**U**〉 + **u** to compute the root-mean-square of the velocity fluctuations *u*_rms_ = 〈**u** ⋅ **u**〉^1/2^, where **u** comprises turbulent and pulse-to-pulse velocity fluctuations [28]. To estimate the shear rate, we computed the scalar

$$\begin{array}{c} \gamma_{3\text{D}}=\varepsilon_{\text{max}}-\varepsilon_{\text{min}} \end{array}$$ (5)

where *ε*_max_ and *ε*_min_ are the maximum and minimum eigenvalues of the rate-of-strain tensor [29], (in matrix form) given as

$$S=\frac{1}{2}\left[\left(\frac{\partial U}{\partial X}\right)+{\left(\frac{\partial U}{\partial X}\right)}^{T}\right]\text{.}$$ (6)

In S1 Fig, the values of *γ*_3*D*_ in the central *Y*-*Z*–slice are compared to the shear rates computed from the 2D velocity field [*V*,*W*] in the same slice. It illustrates that shear rates based on 2D data underestimate the actual shear rates in the field due to non-zero gradients in the out-of-plane velocity field.

## Results

### Pressure and projected orifice area

**Fig 2:**
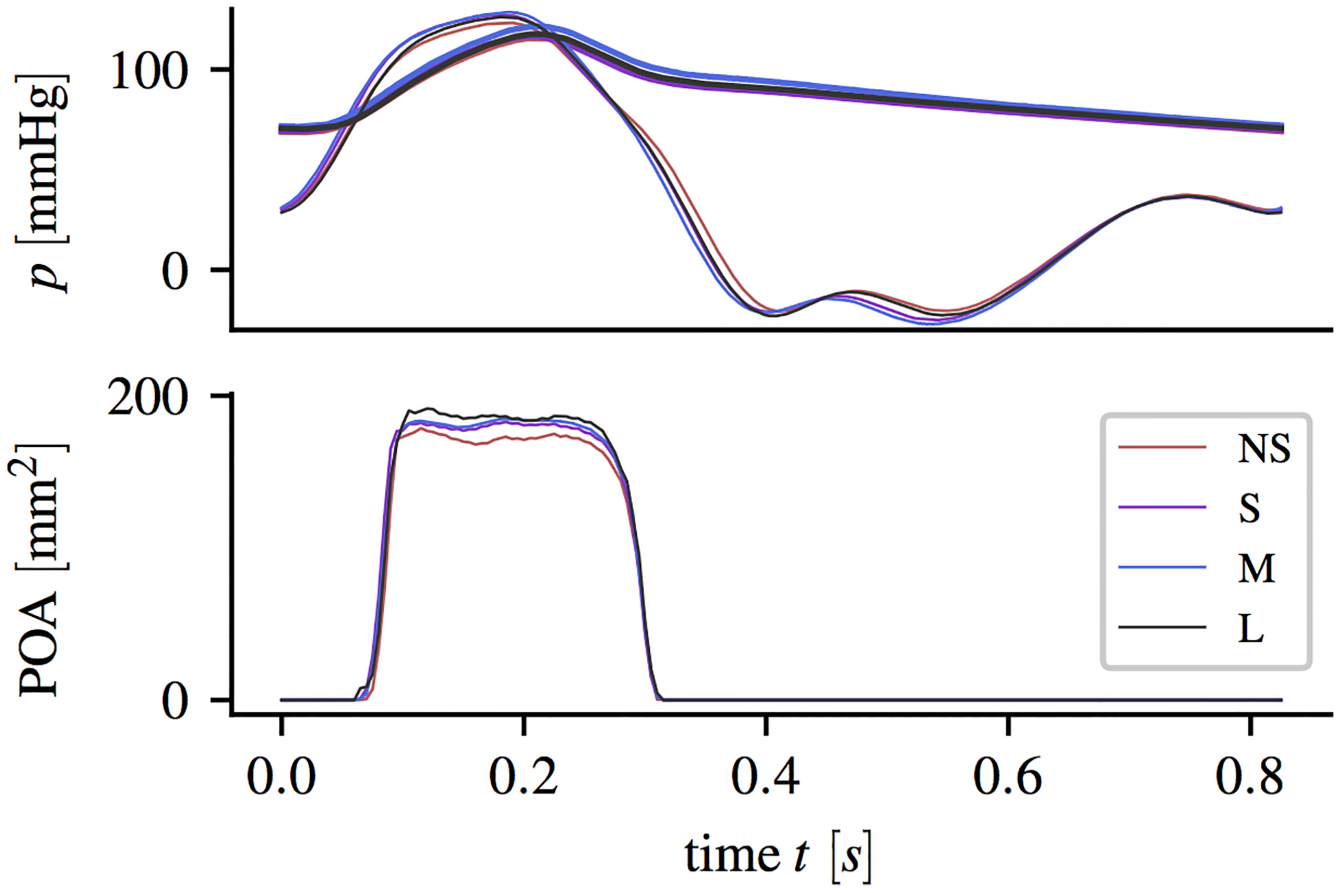
Pressure and projected orifice area. Ventricular pressure *p*_LV_ (solid bold lines) and aortic pressure *p*_a_ (solid thin line) pressure curves (averaged over five subsequent pulses) and projective orifice area POA, measured during experiments with different aortic root phantoms.

Fig 2 shows the left ventricular and the aortic pressure together with the POA (average from five successive pulses) for the different aortic root phantoms.

Small differences in the pressure curves could be mainly assigned to differences in the total flow resistance (different aortic root dimensions and different POA). Ventricular pressure values were negative during early diastole when fluid was accelerated from the tank into the LV-chamber.

Opening started at approximately 60 ms with respect to the pump stroke. The exact opening times differ by less than 5 ms between the aortic root geometries. In all geometries, the whole valve opening process took approximately 30 ms. During mid-systole, the different aortic root showed different POA between 170 and 185 mm^2^. The POA for NS was approximately 15 mm^2^ (8%) smaller than for L. Closing of the valve started at around 240 ms and ended at 300 s. Despite different maximum POA, the closing curves were nearly identical for all aortic root geometries.

### Flow fields

The experimental protocol with 4 phantoms, 13 time points, and 16 repetitions yielded 4 × 13 × 16 = 832 instantaneous velocity fields for the flow past the valve and the same number of fields for the SOV. In the following, mean and instantaneous flow fields at selected instances are presented together with integral quantities (e.g. mean and the fluctuating kinetic energy in specific domains).

#### General behavior of mean flow

**Fig 3:**
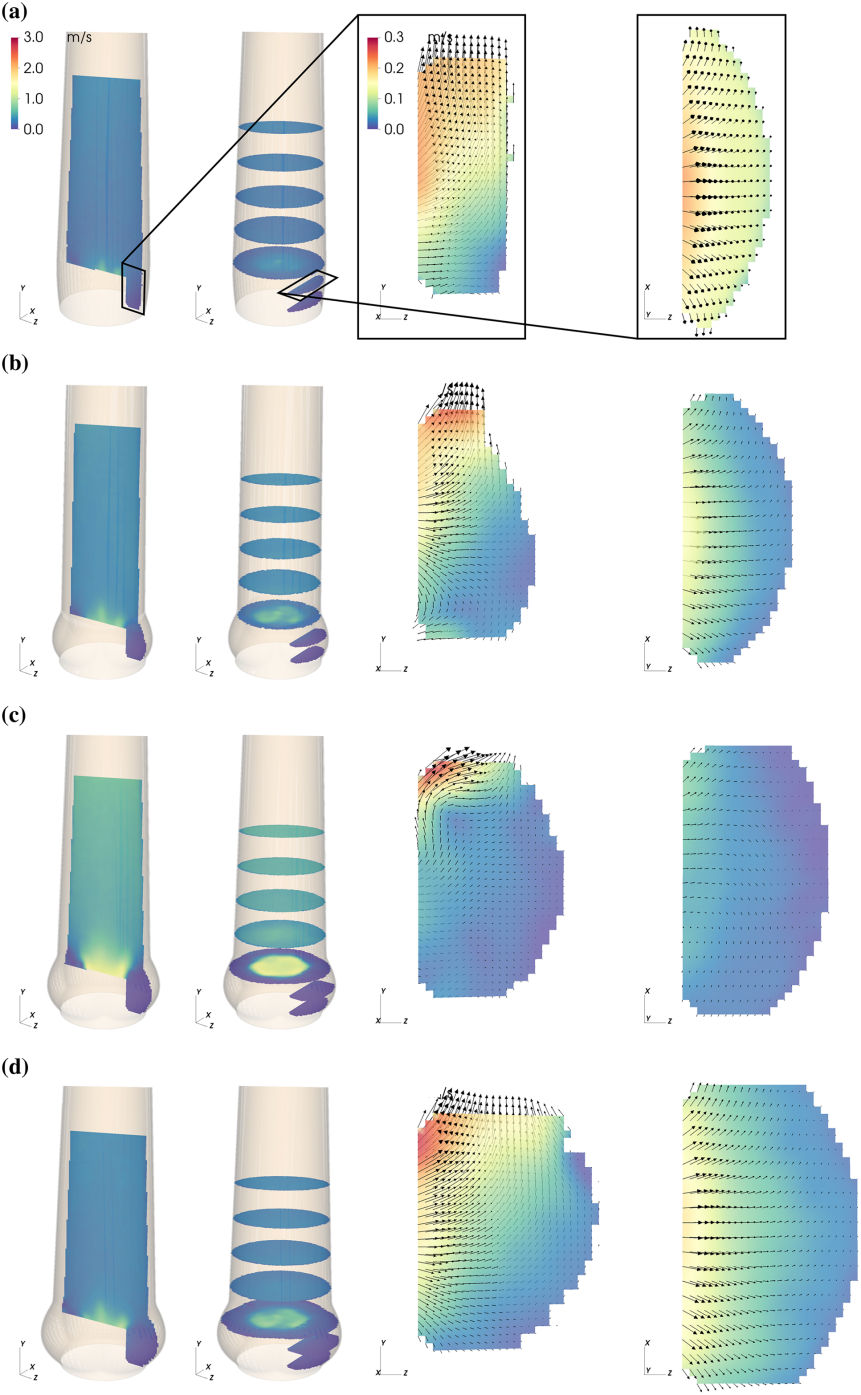
Mean flow fields in the AAo and in the SOV. Mean velocity magnitude |〈**U**〉| in the NS (a), S (b), M (c) and L (d) aortic root configuration during valve opening (*t* = 0.09 s. Each panel (a)—(d) shows axial and cross-sectional slices in the bulk flow downstream of the AVBP and a vertical and horizontal slice centered in the front-facing of the SOV.

**Fig 4:**
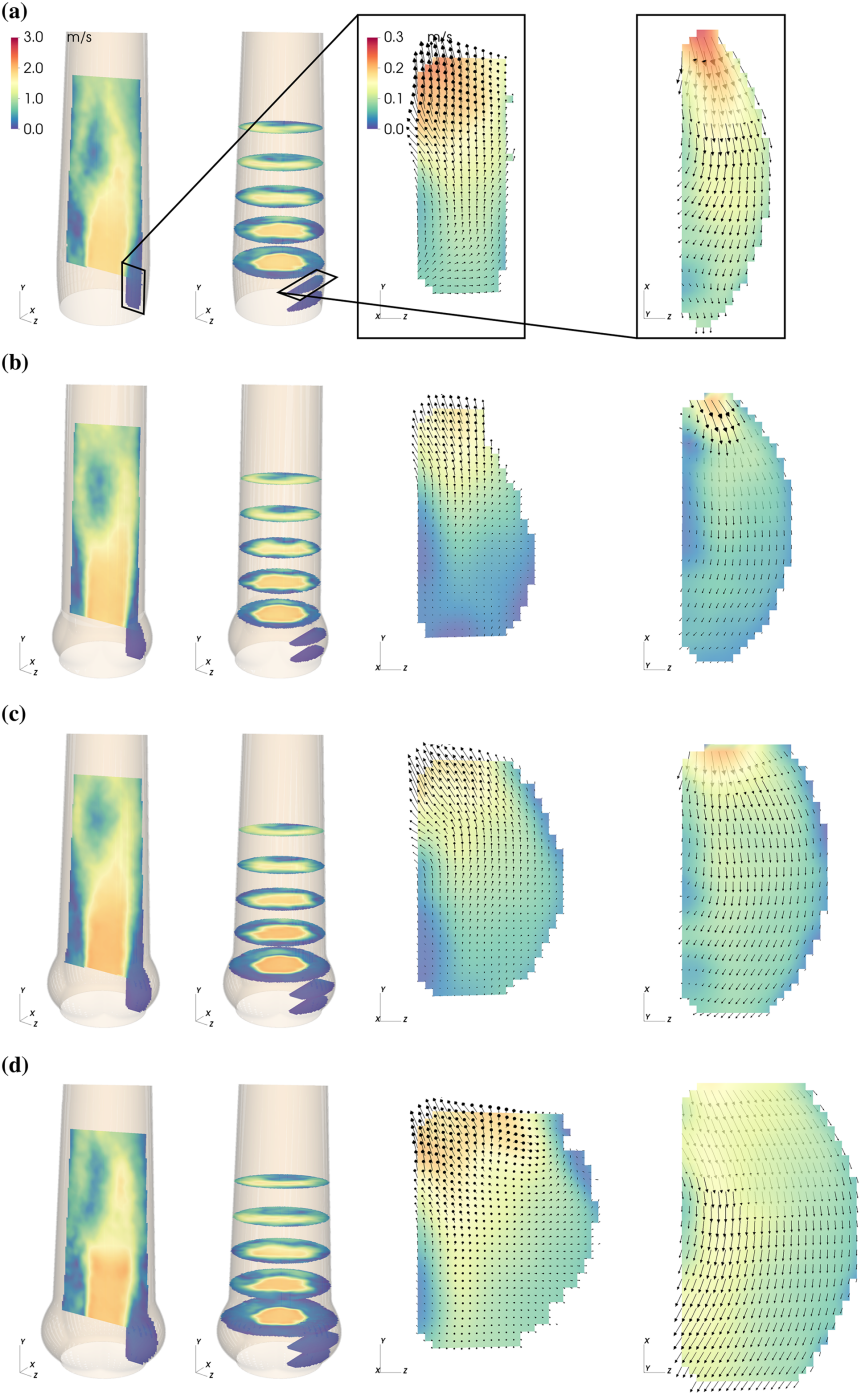
Mean flow fields in the AAo and in the SOV. Mean velocity magnitude |〈**U**〉| in the NS (a), S (b), M (c) and L (d) aortic root configuration during mid-systole (*t* = 0.18 s. Each panel (a)—(d) shows axial and cross-sectiononal slices in the bulk flow downstream of the AVBP and a vertical and horizontal slice centered in the front-facing of the SOV.

**Fig 5:**
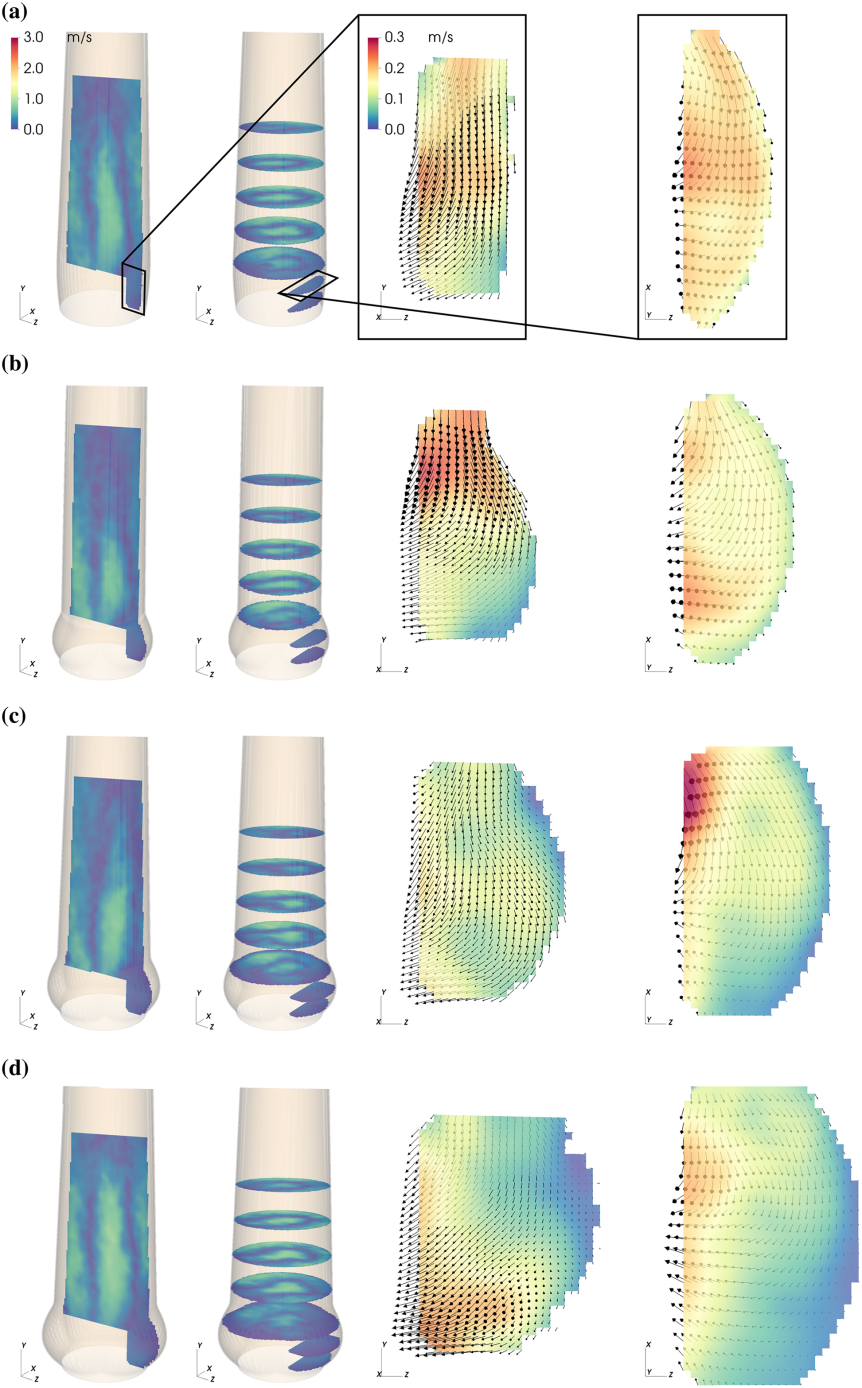
Mean flow fields in the AAo and in the SOV. Mean velocity magnitude |〈**U**〉| in the NS (a), S (b), M (c) and L (d) aortic root configuration during valve closure (*t* = 0.30 s. Each panel (a)—(d) shows axial and cross-sectiononal slices in the bulk flow downstream of the AVBP and a vertical and horizontal slice centered in the front-facing of the SOV.

The magnitude of the mean flow field past the valve and in the sinus is shown in Figs 3–5 for three representative instances: valve opening (*t* = 0.09 s), mid-systole (*t* = 0.18 s), and closing (*t* = 0.30 s).

**Fig 6:**
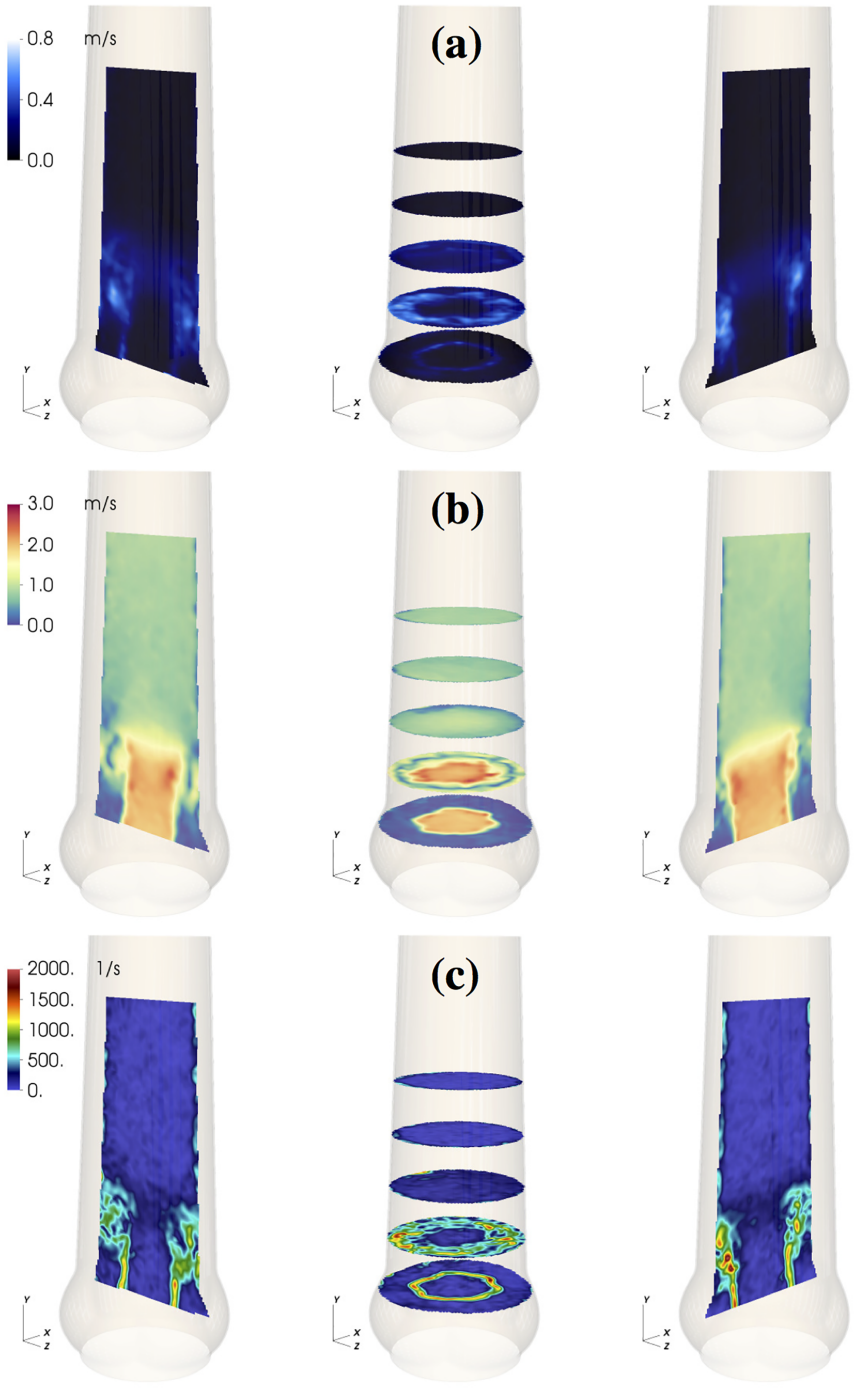
Starting vortex flow fields. Flow field in the medium sized aortic root (M) at *t* = 0.12 s: RMS velocity fluctuations *u*_rms_ (a); instantaneous velocity magnitude field |〈**U**〉| (b); 3D maximum shear rate *γ*_3D_ (c).

During valve opening, fluid in the SOV was pushed in radial direction and out of the sinus (Fig 3, vertical and horizontal slice in the SOV). Instantaneous flow fields at *t* = 0.12 s (Fig 6, see also S1 Video) show a vortex ring approximately one diameter (*d*_a_) past the AVBP. We assign this flow structure to the starting vortex that develops at the tip of the leaflets as described in [30] and [31]. This flow configuration was associated with moderate pulse-to-pulse velocity fluctuations (Fig 6a) and elevated local shear rates (Fig 6c). Because of the distance between the leaflet tips and the vessel wall and the straight geometry of the AAo, the starting vortex did not interact directly with the wall and was advected out of the phantom within approximately 0.05 s. This is different to the (starting) vortices described in [18, 30], which impinge on the wall below the STJ and remain close to the valve for some time during systole.

From early to mid systole, an AJ of high-momentum fluid with a flat velocity profile penetrated the AAo (Fig 4). Flow instabilities developed along the AJ and led to turbulent flow. The laminar potential core of the AJ reached a length of approximately 2*d*_a_ with velocities of about 2 m/s at peak-systole (*t* = 0.21 s).

**Fig 7:**
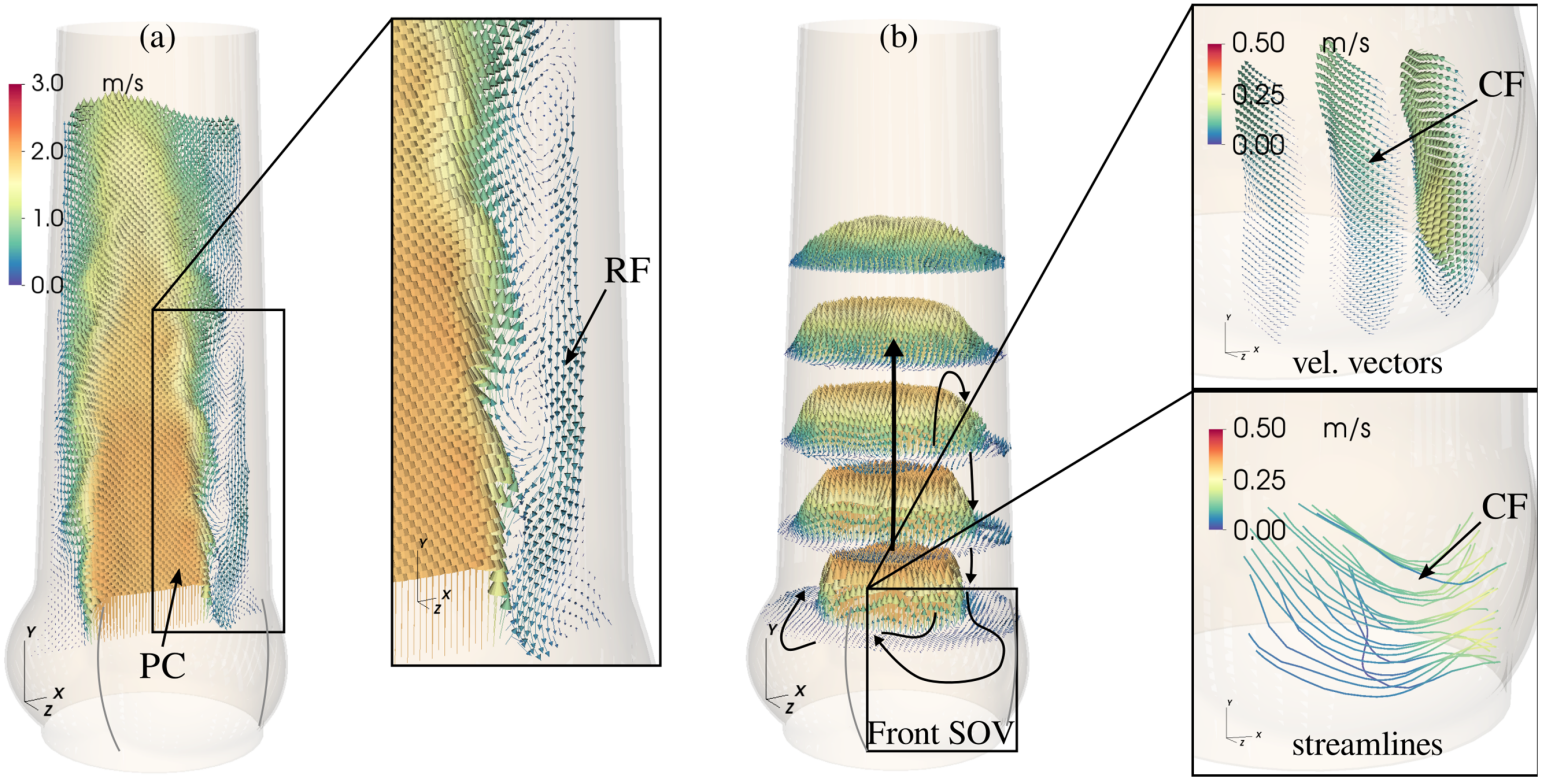
Main flow structures. General organization of the mid-systolic flow shown for the results obtained in M. Mean velocity field 〈**U**〉 in the *XY*-plane (a) and in the cross-sectional *XZ*-plane (b) past the valve at peak systole. The mean velocity field in the front-facing SOV (aligned with positive *Z*-axis) for the same instance is depicted in the enlarged images to the right (velocity vectors and streamlines). Notice, that the orientation of the flow field (i.e. its reference frame) is the same for all representations. Indicated are the potential core (PC), the retrograde flow (RF) near the wall, and the circumferential flow (CF) pattern in the SOV.

At the same time, fluid between AJ and AAo wall was pushed upstream toward the SOV (retrograde flow, RF). This process was asymmetric in the sense that fluid is primarily entering the SOV portion in the back (Fig 7, see also S2 Video). The RF led to a relatively strong circumferential flow within the SOV (Fig 4, vertical and horizontal slice in the SOV). In general, the velocity magnitudes in the SOV remained below 15% of the mean flow velocities.

Toward the end of systole, the AJ decelerated while the RF increased. At *t* = 0.30 s the valve closed with RF entering the whole SOV. Within the SOV a radial flow toward the center was present for all aortic root sizes. Fig 2 and visual inspection of the leaflet motion captured with the axial camera confirm that the corresponding valve leaflet is still in motion. Therefore, we suspect that this radial flow is directly connected to the closing motion of the valve leaflet.

The main flow structures are schematically summarized in Fig 7, including the central AJ with the potential core and the asymmetric RF leading to circumferential flow within the SOV.

#### Effect of aortic root size

In the following the term *AAo flow* will be used for the flow in the AAo strictly downstream of the AVBP, and the term *SOV flow* will be used to describe the flow behind the prosthesis leaflet in the SOV. Results are compared in two groups: (i) among the scaled aortic root phantoms with increasing AAo and SOV size (S, M, L) and (ii) between the two aortic root phantoms with different SOV size but equal AAo lumen (NS, M).

**Fig 8:**
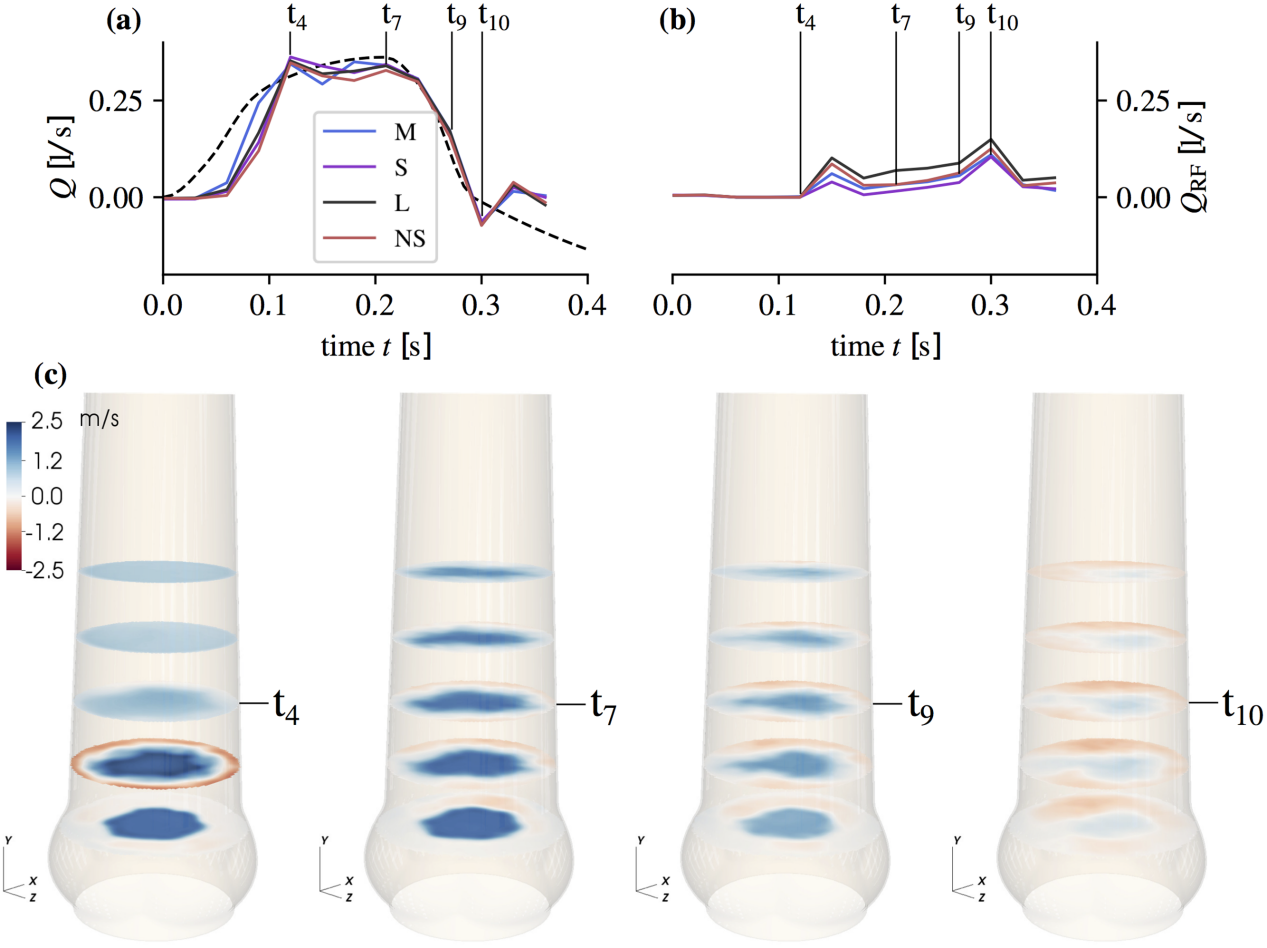
Flow rates and mean streamwise flow field. Net flow rate (a) and RF flow rate (b) (computed form 〈*U*_*y*_〉 at *Y* = 0) at the time points *t*_*j*_ = 0.0, 0.03, …, 0.36 s together with the piston pump induced flow rate (dashed line). Mean streamwise velocity component |〈*U*_*y*_〉| (c) in aortic root size M after valve opening (*t*_4_ = 0.12 s), at peak-systole (*t*_7_ = 0.21 s), during flow deceleration (*t*_9_ = 0.27 s), and during valve closing (*t*_10_ = 0.30 s). Cross-sections are shown at *Y* = −20, −10, 0, 10, 20 mm.

**AAo flow**: Fig 8 shows velocity fields for the axial component 〈*U*_*y*_〉 in M together with net flow rates and RF rates for all geometries. The net flow rates were calculated by numerical integration of 〈*U*_*y*_〉 over the cross-section at *Y* = 0. For the RF rates, only the negative mean streamwise velocities were integrated.

**Fig 9:**
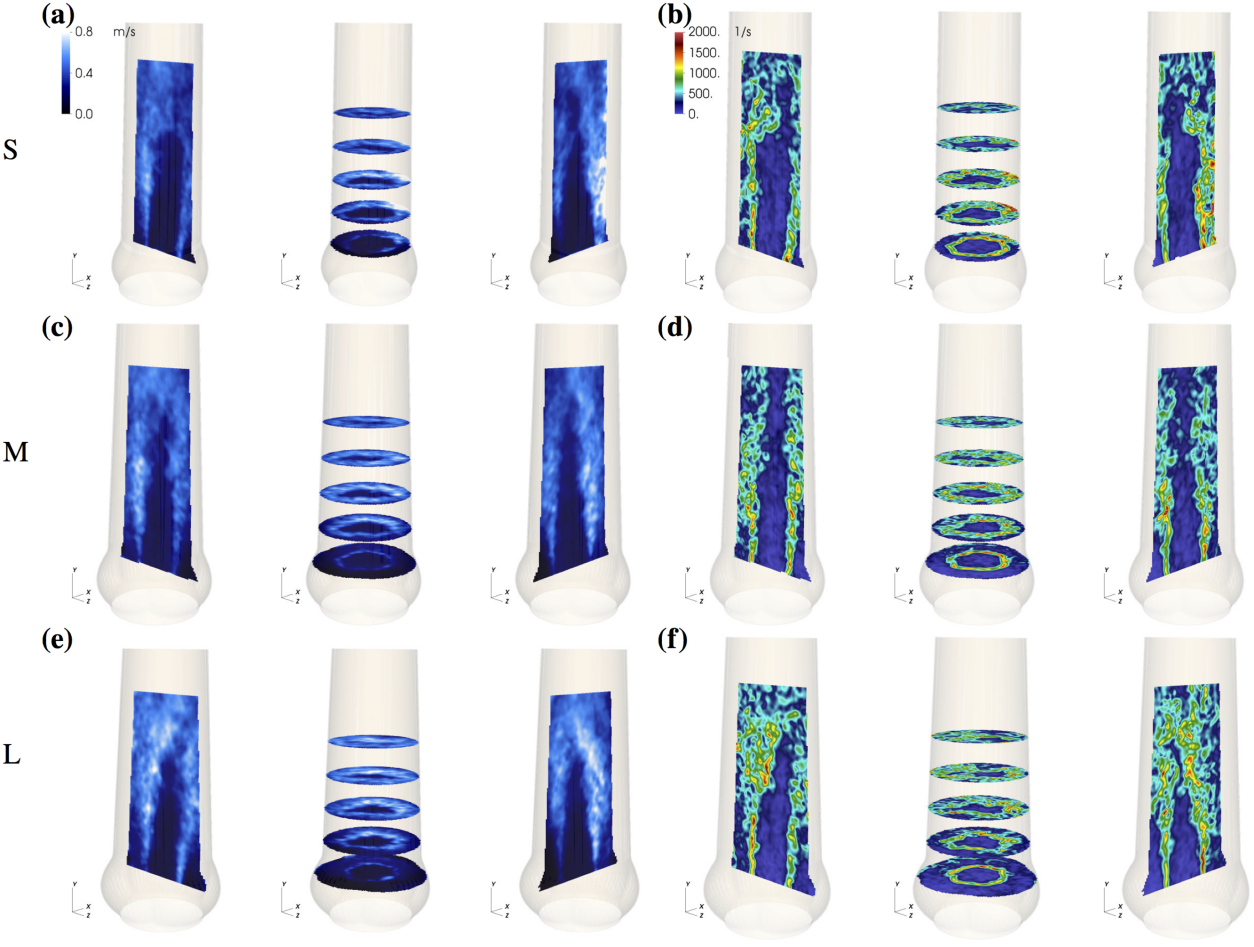
Properties of aortic jet shear layer. RMS velocity fluctuation field *u*_rms_ (a, c, e) and 3D shear rate *γ*_3D_ (b, d, f) at *t* = 0.21 s for small (S), medium (M), and large (L) aortic root size.

The starting vortex (Fig 8 at *t*_4_) led to local axisymmetric retrograde flow close to the AAo wall. Differences between the different aortic root phantoms were small. During mid-systole (*t*_7,9_), asymmetric RF is established with most flow going toward the SOV portion in the back. At valve closing (*t*_10_), RF reaches its maximum value for all aortic root phantoms. Throughout the whole pulse, the magnitude of RF was highest in L. The RF rate in M and NS were nearly the same during mid-systole and S had the lowest RF rate. Fluctuations and shear rates in the AAo during mid-systole (Fig 9) were highest in L, and the potential core was shorter than in S and M.

**SOV flow**: During valve opening, leaflet motion induced radial flow within the SOV (Fig 3 and S2 Fig). The fluid that was displaced by the leaflets led to an antegrade flow out of the SOV, which is slowest in L probably due to the wider distance between leaflet tip and STJ (Fig 3d). The situation is somewhat different in M (Fig 3c) where we suspect that the valve opened a little bit earlier such that this flow field shows the beginning of the starting vortex at the leaflet tip.

During mid-systole (Fig 4), circumferential flow was dominant. In L it affected almost all fluid in the SOV portion, while it did not reach to the base of the SOV in S. In contrast, the peak circumferential velocities were highest in the smaller SOV (S and NS).

Leaflet motion during valve closure (Fig 5) caused radial flow toward the center of the aortic root. The valve closing volume was compensated by an inflow into the SOV portion. In S, this inflow led to high retrograde velocities close to the STJ due to the small gap between leaflet tip and STJ. In the other roots, most of the closing volume was compensated by circumferential inflow fed by RF into the back SOV portion.

**Integral quantities**: From the velocity fields 〈**U**〉 and *u*_rms_, the mean and the fluctuating kinetic energy were computed for a given domain Ω as

$$\begin{array}{c} E_{\text{MK}}^{\Omega }=\frac{\rho }{2}\int_{\Omega }{\left|\left\langle U\right\rangle \right|}^{2}\;dV\;\text{,}\;E_{\text{FK}}^{\Omega }=\frac{\rho }{2}\int_{\Omega }u_{\text{rms}}^{2}\;dV \end{array}$$ (7)

**Fig 10:**
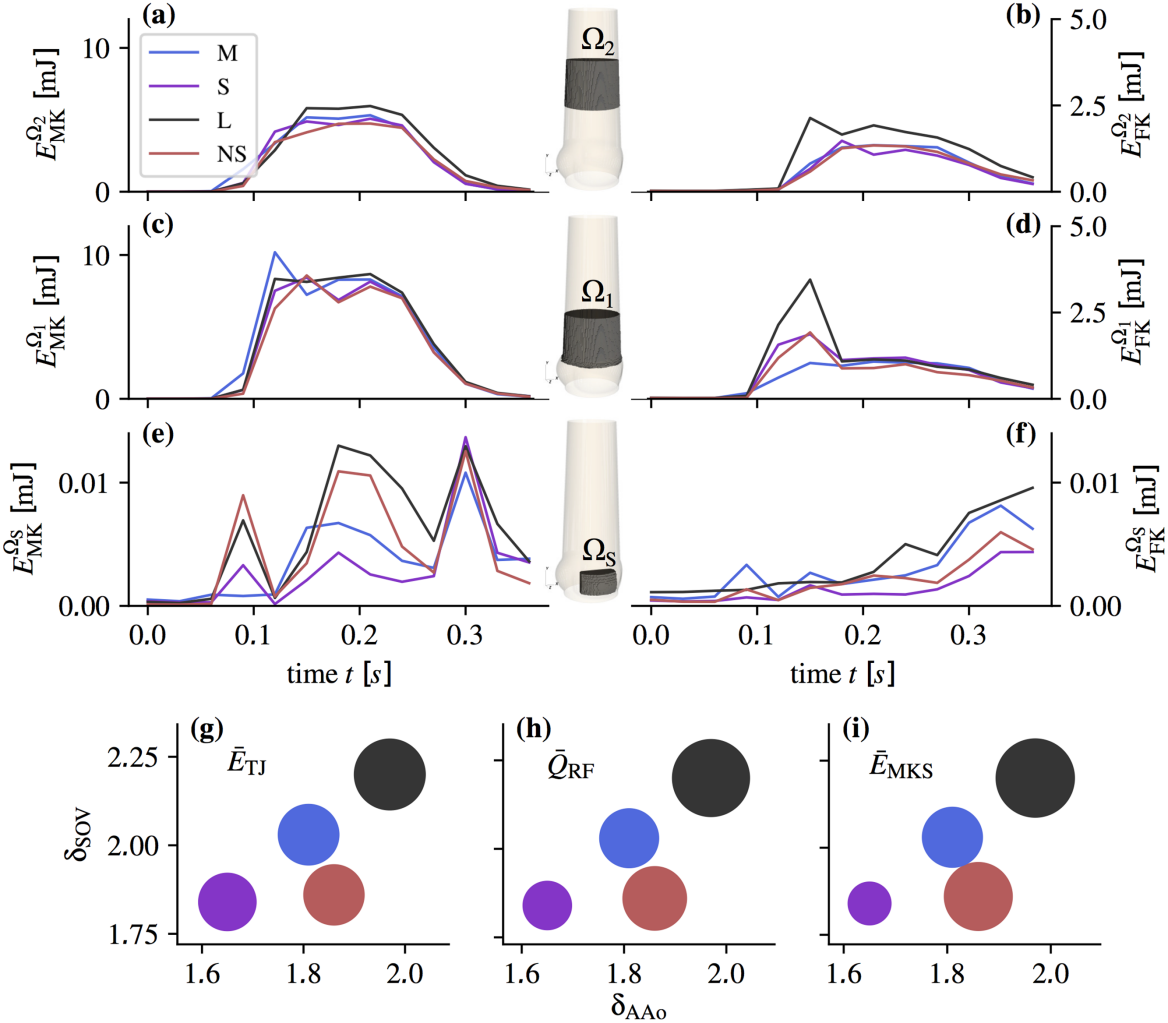
Integral quantities. Above: Mean kinetic energy $E_{\text{MK}}^{\Omega }$ (a, c, e) and fluctuating kinetic energy $E_{\text{FK}}^{\Omega }$ (b, d, f) in function of time for the domains Ω_1_, Ω_2_ past the valve and the sinus flow domain Ω_S_ (as indicated in the aortic root sketches in the center). Note the different scales for (e) and (f). Below: Scatter plots of integrals ${\bar{E}}_{\text{TJ}}$ (g), ${\bar{Q}}_{\text{RF}}$ (h), and ${\bar{E}}_{\text{MKS}}$ (i) in function of the *δ*_SOV_ and *δ*_AAo_ (NS red, S violet, M blue, L black) with the sizes of circles corresponding to the parameter integral values calculated using Eq (8)).

We defined three domains (Fig 10): the sinus domain Ω_S_, and two domains Ω_1_ and Ω_2_ in the AAo extending from 0 to *d*_a_ and from *d*_a_ to 2*d*_a_, respectively, above the tip of the valve.

Fig 10a–10f shows the evolution of these energies throughout the pulse. In the AAo domains we identified two phases: the acceleration phase (0.06 < *t* < 0.18 s) with the starting vortex and the mid-systolic and deceleration phase (0.18 ≤ *t* < 0.36 s) dominated by a turbulent shear layer and RF. The fluctuating kinetic energy peaked during the acceleration phase when the starting vortex dominated the flow field. During mid-systole, the AJ was fully developed and featured a transitional to turbulent shear layer. In Ω_1_, the fluctuating kinetic energy was nearly the same in all aortic root phantoms. In Ω_2_, the fluctuating kinetic energy increased in all phantoms indicating a higher level of turbulent fluctuations. Remarkably, the increase in L was significantly stronger than in the other phantoms.

In the SOV domain Ω_S_, three phases could be identified: valve opening, mid-systole, and valve closure are reflected by three distinct peaks in the mean kinetic energy $E_{\text{MK}}^{\Omega_{S}}$ in all aortic root. During mid-systole, the mean kinetic energy was highest in L, followed by NS, M and S. The fluctuating kinetic energies in the SOV increased during the pulse and reached the highest values after valve closing. This increase was most significant in L.

To better evaluate the effect of SOV size and AAo lumen, we defined normalized diameters for the SOV, *δ*_SOV_ = (*r*_co_ + *r*_s_)/*d*_jet_, and for the AAo, *δ*_AAo_ = 2 *r*_stj_/*d*_jet_. For normalization we used the jet diameter $d_{\text{jet}}=\sqrt{4\;A_{\text{POA}}/\pi }$ where *A*_POA_ is the mean POA during systole (Fig 2). Furthermore, we defined metrics in specific regions and time periods (Eq (8)), where distinct dynamics are present in the flow: ${\bar{E}}_{\text{TJ}}$, quantifies the turbulence intensity of the fully turbulent flow past the valve after transient phenomena related to AJ establishment have been washed out; ${\bar{Q}}_{\text{RF}}$ quantifies the negative flow rate in the mid-systolic phase excluding times during which valve opening and closing affects the flow field; and ${\bar{E}}_{\text{MKS}}$ quantifies the flow activity in the SOV as an estimate of the sinus washout efficiency.

$$\begin{array}{c} {\bar{E}}_{\text{TJ}}=\frac{1}{t_{b}-t_{a}}\int_{t_{a}}^{t_{b}}E_{\text{FK}}^{\Omega_{2}}dt\;\text{with}\;t_{a}=0.18\;s,\;t_{b}=0.36\;s \end{array}$$ (8a)

$$\begin{array}{c} {\bar{Q}}_{\text{RF}}=\frac{1}{t_{b}-t_{a}}\int_{t_{a}}^{t_{b}}Q_{\text{RF}}^{\Omega_{2}}dt\;\text{with}\;t_{a}=0.15\;s,\;t_{b}=0.30\;s \end{array}$$ (8b)

$$\begin{array}{c} {\bar{E}}_{\text{MKS}}=\frac{1}{t_{b}-t_{a}}\int_{t_{a}}^{t_{b}}E_{\text{MK}}^{\Omega_{S}}dt\;\text{with}\;t_{a}=0.12\;s,\;t_{b}=0.27\;s \end{array}$$ (8c)

These definitions are based on a qualitative assessment of the evolution of the mean velocity and RMS velocity fluctuation fields. They are specific to the present experiments and might not be applicable in general, because the dynamics of the flow strongly depends on the given configuration.

Fig 10g–10i shows the magnitudes of ${\bar{E}}_{\text{TJ}}$, ${\bar{Q}}_{\text{RF}}$, and ${\bar{E}}_{\text{MKS}}$ in function of *δ*_SOV_ and *δ*_AAo_. All quantities increased with *δ*_AAo_. The situation is less clear for *δ*_SOV_: A comparison of the results for M and NS indicates that *δ*_SOV_ has an effect on RF and SOV flow, ${\bar{Q}}_{\text{RF}}$ and ${\bar{E}}_{\text{MKS}}$ and that it has no effect on turbulence intensity ${\bar{E}}_{\text{TJ}}$.

## Discussion

Systolic flow in the aortic root is dominated by the AJ, which is directly related to other flow phenomena such as a starting vortex, turbulent flow along the AJ shear layer, RF near the AAo wall, and vortical flow in the SOV. In the following, we discuss the character and intensity of these phenomena as a function of the aortic root geometry.

### Starting vortex

At the beginning of systole, a starting vortex develops at the leaflet tips. Shear rates associated with this flow structure reach relatively high values (∼1500 s^−1^) in single spots in the shear layer but otherwise stay below 1000 s^−1^ (Fig 6) and the corresponding shear stresses remain below the threshold for platelet activation [32]. Stronger pulse-to-pulse velocity fluctuations and higher shear rates were observed in the NS, S, and L, compared to M (see $E_{\text{FK}}^{\Omega_{1}}$ at *t* = 0.12 s in Fig 10d).

At valve opening, leaflet motion induces flow in the SOV. The organization of this flow is sensitive to the local geometry. Fig 3 shows that at *t* = 0.09 s considerably more fluid is flowing out of the SOV in NS than in M. We speculate that this increased outflow perturbs the roll-up of the starting vortex in NS causing higher levels of fluctuation and shear.

These observations suggest that the SOV geometry plays a delicate role during valve opening. In a comparable sinus-less aortic root configuration, Toninato *et al* (2016) described similar phenomena: The starting vortex was advected downstream and altered the flow past the valve, which they related to a reduction of the AJ diameter (diameter of *vena contracta*) [19]. This also compares well to the reduced POA for NS (Fig 2).

### Turbulent flow in the AAo

A turbulent shear layer forms between the fast AJ and the slow fluid near the vessel wall (Fig 9). The closer the AJ is to the AAo wall, the more the shear layer is affected by the confining wall. For small AAo, the shear layer becomes a boundary layer. For large AAo, we have a free shear layer. According to hydrodynamic stability theory, this has a direct effect on the shear layer dynamics because wall-bounded flows tend to be more stable than free shear layers [33].

In our experiments, even the smallest aortic root had an AAo lumen, which was larger than the AJ such that free shear layers were present in all cases (1.6 < *δ*_AAo_ < 2). We expect this to be different in the native healthy case, where the AJ typically occupies most of the AAo [15, 17].

Until one diameter *d*_a_ downstream of the valvular orifice, the unstable shear layers develop similarly in all geometries and they remain rather thin (Fig 9). Accordingly, the fluctuating energy in Ω_1_ (after the starting vortex is advected downstream) is nearly the same for all aortic root (Fig 10d). Further downstream, the flow exhibits stronger turbulent fluctuations (Fig 10b). This growth is most pronounced in L, which shows also the highest increase in fluctuating kinetic energy from Ω_1_ to Ω_2_. Accordingly, we also find higher shear rates in L (Fig 9f). We speculate that (as the shear layers become thicker in Ω_2_) the stabilizing effect of the wall becomes apparent in NS, S and M, but not so much in L.

This results in an AAo turbulence intensity ${\bar{E}}_{\text{TJ}}$ which appears to scale with *δ*_AAo_ (Fig 10). At the same time, we should clarify that this observation is for our *in-vitro* configuration where the jet is parallel to the straight vessel wall. In the native case, the curvature of the aorta causes skewed velocity profiles and strong helical and retrograde flow in the AAo [16, 17]. This will certainly affect the AJ stability and turbulence levels. Nevertheless, the stabilizing effect of smaller AAo diameters may still be present.

### Retrograde flow near the wall

Another remarkable phenomenon is the RF near the AAo wall transporting fluid toward the SOV. Similar flow phenomena have also been reported for native AV [15] and AV prostheses [34]. In their oversized configuration (comparable to M), Toninato (2016) *et al*. reported RF leading to increased energy losses and affecting the flow pattern in the SOV [19].

The flow over a backward-facing step can serve as a simple model for the basic mechanisms leading to RF. This canonical flow problem exhibits a recirculation region downstream of a backward-facing step (corresponding to the tip of the valve leaflets). The recirculation region is limited by a stagnation point where the pressure has a local maximum. This results in a positive pressure gradient near the wall, which induces retrograde flow toward the step.

In the aortic root, the situation is more complex due to flow instabilities and turbulence in the AJ which lead to unsteady flow reattachment on the AAo wall. Nevertheless, the basic mechanism leading to recirculation along the AAo wall persists. Furthermore, flow pulsatility leads to an additional positive pressure gradient during late systolic flow deceleration [34] which can be held accountable for the increase in *Q*_RF_ from *t*_7_ to *t*_9_ (Fig 8b).

In summary, the observed RF can be explained by two fundamental phenomena: (i) reattachment of the AJ leading to a stagnation point at the AAo wall with a local pressure maximum and (ii) positive pressure gradient due to flow deceleration in late systole. Both phenomena create a situation where the fluid between AJ and AAo wall is pushed in upstream direction.

The bigger the relative AAo diameter *δ*_AAo_, the more low-momentum fluid is available in the AAo, which explains why the RF rate increases from S to M to L (see ${\bar{Q}}_{\text{RF}}$ in Fig 10h). Conversely, the nearly identical flow rates in late systole from M and NS confirms that SOV size barely affects RF.

RF is not axisymmetrically distributed along the AAo wall. This is probably due to mass conservation, which leads to concentrated RF into one SOV portion (in the back of the flow fields at *t*_7_ and *t*_9_ in Fig 8c) and slow antegrade flow out of the other two SOV portions.

RF near the AAo wall has several consequences for valve function. Fluid particles might be trapped in the recirculation region, causing increased residence time. Together with the exposure to moderate to high shear stresses from the starting vortex and jet shear layer, this may be a factor in thrombus formation. At the same time, RF could enhance the fluid turnover in the SOV and thereby support wash-out.

### SOV flow

In general, SOV flow strongly depends on the whole flow field past the AV, as demonstrated by Markl (2005) *et al* in an *in-vivo* study [16]. It can therefore be expected that asymmetric velocity profiles and helical flows due to aortic curvature have additional effects on the sinus flow, which were not captured in the present study. In our experimental setting, SOV flow appears to be dominated by two different phenomena: (i) leaflet motion during opening and closing, (ii) RF due to AJ reattachment at the AAo wall.

Impingement of the AJ below the STJ was not observed because all aortic root phantoms had an STJ diameter that was larger than the diameter of the AJ such that the jet flow always reattached above the STJ. The AJ partly impinges on the wall below the STJ when the AAo is small, when the prosthesis is slightly tilted, or when the AJ is rather wide or divergent due to the specific prosthetic valve design (e.g. [35, 36]) In this case, a substantial amount of momentum is transported directly from the AJ into the SOV leading to vortical flow structures in the SOV as reported by Moore *et al*. (2014) [18].

During mid-systole, the mean velocity fields in the SOV at *t* = 0.18 s (Fig 4b, 4c and 4d) and ${\bar{Q}}_{\text{RF}}$ and ${\bar{E}}_{\text{MKS}}$ (Fig 10h and 10i) for S, M, and L suggest that SOV flow becomes stronger for larger aortic root because more RF reaches the SOV. At the same time, we observe that the mean kinetic energy in the SOV, ${\bar{E}}_{\text{MKS}}$, is higher in NS than in M (although ${\bar{Q}}_{\text{RF}}$ is nearly the same), which might be attributed to the fact that the same volume of RF has to be distributed in a smaller SOV volume leading to higher flow velocities. We conclude that large AAo lumen and (to a lesser extent) small SOV size can contribute to higher flow velocities in the SOV, which might be beneficial for SOV washout lowering the risk of thrombus formation. We were unable to detect any regions of stagnant flow during a heart cycle. Nevertheless, the magnitude of SOV flow velocities remained rather low in all experiments (5–10% of the AJ velocities). This is comparable to the *optimal surgical configuration* reported by Toninato (2016) *et al* [19].

The SOV flow topology in the present experiments is more complex than in the classical ASV first described by Bellhouse and Talbot (1969) [11]. Fluid is pushed into the SOV by the RF as depicted in Fig 7, where it is redistributed in circumferential direction and then pushed out again in forward direction. The redistribution within the SOV can be seen as circumferential flow at mid-height of the front SOV (Fig 4). Under the given measurement conditions, this flow topology persists independent of SOV size.

Finally, we found that larger SOV yielded larger POA (comparing POA for NS and M in Fig 2), which can be associated with lower trans-valvular pressure gradients. This finding is in accordance with other studies [19, 37].

## Limitations

The present study is limited by the use of a simplified geometry for the aortic root with a straight part representing the AAo and symmetric geometries for the three portions of the SOV. In reality, the curvature of the aorta and the difference in size and shape among the three SOV portions are expected to have a significant effect on the 3D flow topology, e.g. in form of secondary, helical flows (as described in [15]). The reattachment of the flow and the particular organization of RF patterns might be different in a curved aorta. However, we expect that even in a real aorta the dimension of the AAo (relative to the aortic jet diameter) to be responsible for the amount of low velocity fluid in the vicinity of the vessel wall. In that case, the relation between free shear flow, RF and *δ*_AAo_ will remain as described above.

Further limitations of the study are related to the material properties of the silicone phantom and of the blood analog fluid. The silicone phantom is approximately three times stiffer than a healthy aorta, and the kinematic viscosity of the blood analog RIM fluid is approximately 15% higher than the apparent kinematic viscosity of blood. Nevertheless, the resulting Reynolds number, Eq (3), remain in a realistic range for all experiments.

## Conclusion

In this study, we analyzed the velocity field past a bio-prosthetic valve in function of the aortic root geometry. Immediately after valve opening, we observed the development of a starting vortex that locally created elevated shear rates. The starting vortex was advected downstream for all aortic root sizes. Along the shear layer of the AJ we found turbulent flow. Turbulence intensity decreased for smaller AAo diameters because the wall appeared to have a stabilizing effect on the AJ shear layers, supporting more favorable flow conditions with lower shear stress and lower energy loss. Between the established AJ and the AAo wall, we observed RF toward the SOV, which was higher for larger AAo diameters. Therefore, the AAo diameter (relative to the AJ diameter) can be identified as the principal determinant for RF and turbulence in the AAo. It also affected the magnitude of SOV flow because RF appeared to be the main driving factor for SOV flow during mid-systole. Furthermore, we found that the mean kinetic energy in the SOV was higher for a smaller SOV (if the AAo diameter was held constant).

The classical flow topology in the SOV with persisting vortices (ASV) could not be detected in any of the experiments. This could be explained by the rather large AAo diameters in our aortic root phantoms, which are typical in patients with aortic stenosis. Therefore, the classical concept of the ASV should be revisited to account for larger AAo. It can be further hypothesized, that SOV flow and its wash-out properties depend strongly on the magnitude and the location of RF, especially in cases where the classical ASV is not present due to a small valve orifice, straight AJ, and bigger cross-section of the aortic root, as often the case after AV replacement.

Finally, the findings of this study indicate that the aortic root geometry might have an effect on aortic valve performance and related adverse events. It suggests that also AAo diameter and SOV size should be considered when selecting a valve replacement therapy for a given patient.

## Supporting information

S1 Appendix Tomographic PIV processing. (PDF)

S1 Fig Comparison of shear rate calculation. Above: Maximum shear rate *γ*_3*D*_ (as defined in Eq 5) at *X* = 0 and maximum shear rate *γ*_2*D*_ derived from 2D velocity field [*V*, *W*] at the same location. Below: Histogram of *γ*_3*D*_ and *γ*_2*D*_.
(PDF)

S2 Fig Flow topology in the sinus of Valsalva. Sinus flow mean velocity magnitude in the non-sinus (NS), the small (S), the medium (M), and the large (L) aortic root configuration, during valve opening (t = 0.09), mid-systole (t = 0.18), and valve closure (t = 0.30).
(PNG)

S1 Video Evolution of the mean flow in the AAo. (MOV)

S2 Video Streamlines of the mean velocity field at peak flow (*t* = 0.21 s). (AVI)
