# Supplementary material for: Three-dimensional flow structures past a bio-prosthetic valve in an in-vitro model of the aortic root
Source: PLoS One. 2018 Mar 16;13(3):e0194384. doi: 10.1371/journal.pone.0194384 (PMC5856406; doi:10.1371/journal.pone.0194384)
Supplement: S1 Appendix — (PDF) [file pone.0194384.s001.pdf]

Tomographic PIV

The pulse delay was set to $\Delta t = 200$ μs for the aortic flow measurements (downstream of the AVBP) and to $\Delta t = 1000$ μs for the SOV measurements where lower velocities were expected. The seeding density of fluorescent particles was approximately 10^3 part./cm^3 corresponding to a particle image density of $0.1 - 0.15$ particles per pixel. The raw images from the four different viewing angles were pre-processed prior to tomographic reconstruction. For these steps, we used the commercial TOMO PIV package DaVis 8.3 (LaVision GmbH, Göttingen, Germany). The procedure is described in detail in Hasler et al. (2016).[1] In the following, only changes and additional post-processing steps with respect to the former study are outlined.

The final interrogation volume size of a multi-step 3D cross-correlation was 48^3 voxel. The interrogation volume overlap was 75 % and the image scaling was 0.036 mm/pixel, which yielded a velocity field resolution of 0.43 mm. Accurate velocity measurements were limited to a domain approximately 0.5 mm away from the solid wall. Close to the wall and across the interface, bias errors compromised the PIV evaluation. Therefore, we excluded this part of the velocity field from the analysis and deleted spurious velocity vectors outside the aortic domain using the phantom geometry as mask. To this end, the phantom geometry was aligned with respect to the reference frame obtained during camera calibration. This alignment was achieved by means of three flourescent beads which were integrated in the AR phantom at well-defined positions during the casting process. After applying the volume mask, the instantaneous flow fields , $\mathbf{U}(\mathbf{X}, t) = [U_x(\mathbf{X}, t), U_y(\mathbf{X}, t), U_z(\mathbf{X}, t)]$ with $\mathbf{X} = [X, Y, Z]$, comprised approximately $385'000$ (aortic flow), respectively $20'000$ (SOV flow), uniformly distributed 3D velocity vectors.

References

1. Hasler, D., A. Landolt and D. Obrist. Tomographic PIV behind a prosthetic heart valve. Exp Fluids 57(80), 2016.
