# Supplementary figures and images for: Three-dimensional flow structures past a bio-prosthetic valve in an in-vitro model of the aortic root
Source: PLoS One. 2018 Mar 16;13(3):e0194384. doi: 10.1371/journal.pone.0194384 (PMC5856406; doi:10.1371/journal.pone.0194384)

$\gamma_{3D}$  (at  $X = 0$ )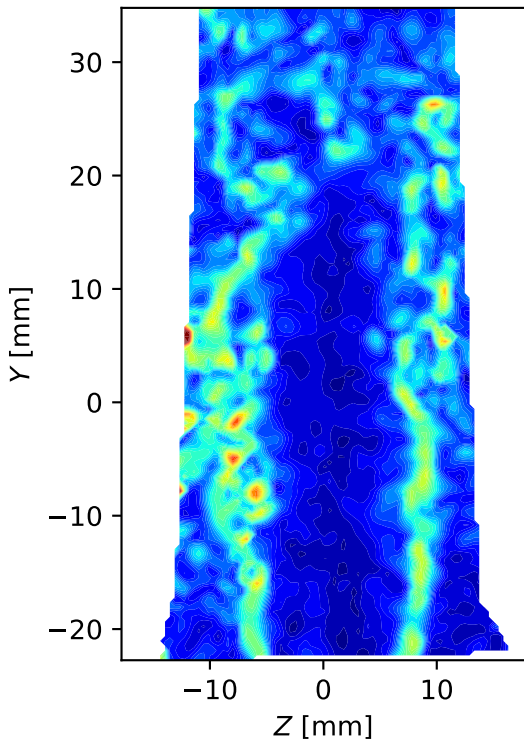 $\gamma_{2D}$  (at  $X = 0$ )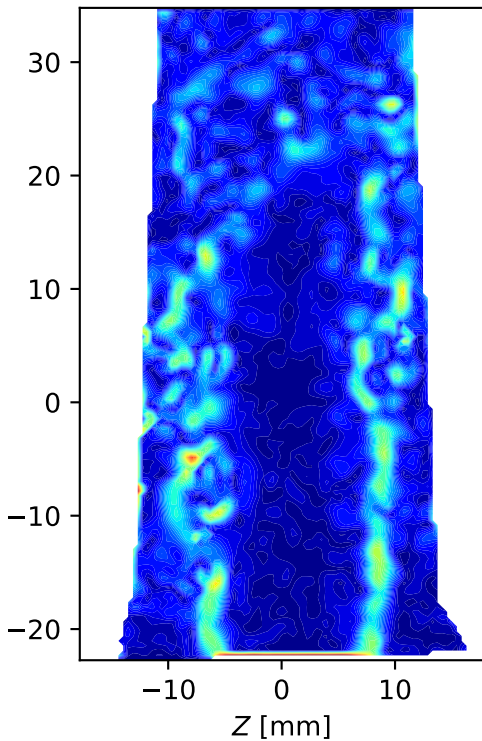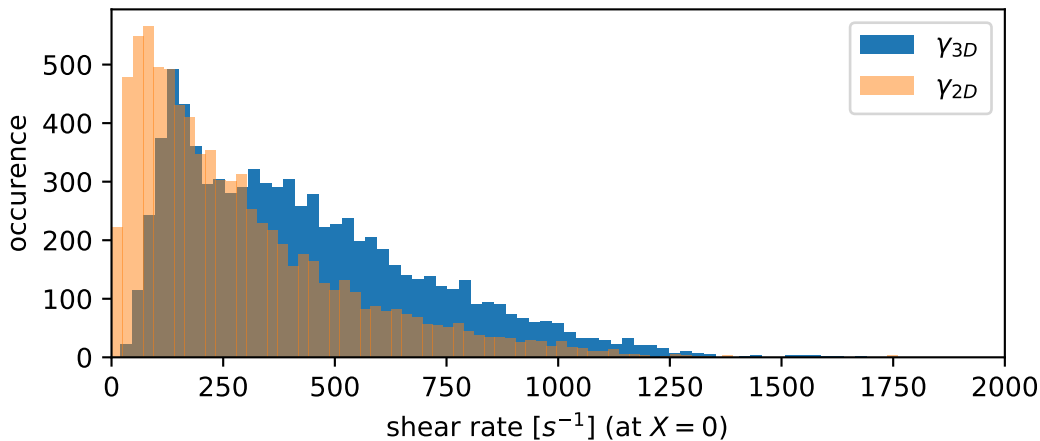

Supplement: S1 Fig — Above: Maximum shear rate γ3D (as defined in Eq 5) at X = 0 and maximum shear rate γ2D derived from 2D velocity field [V, W] at the same location. Below: Histogram of γ3D and γ2D. (PDF) [file pone.0194384.s002.pdf]

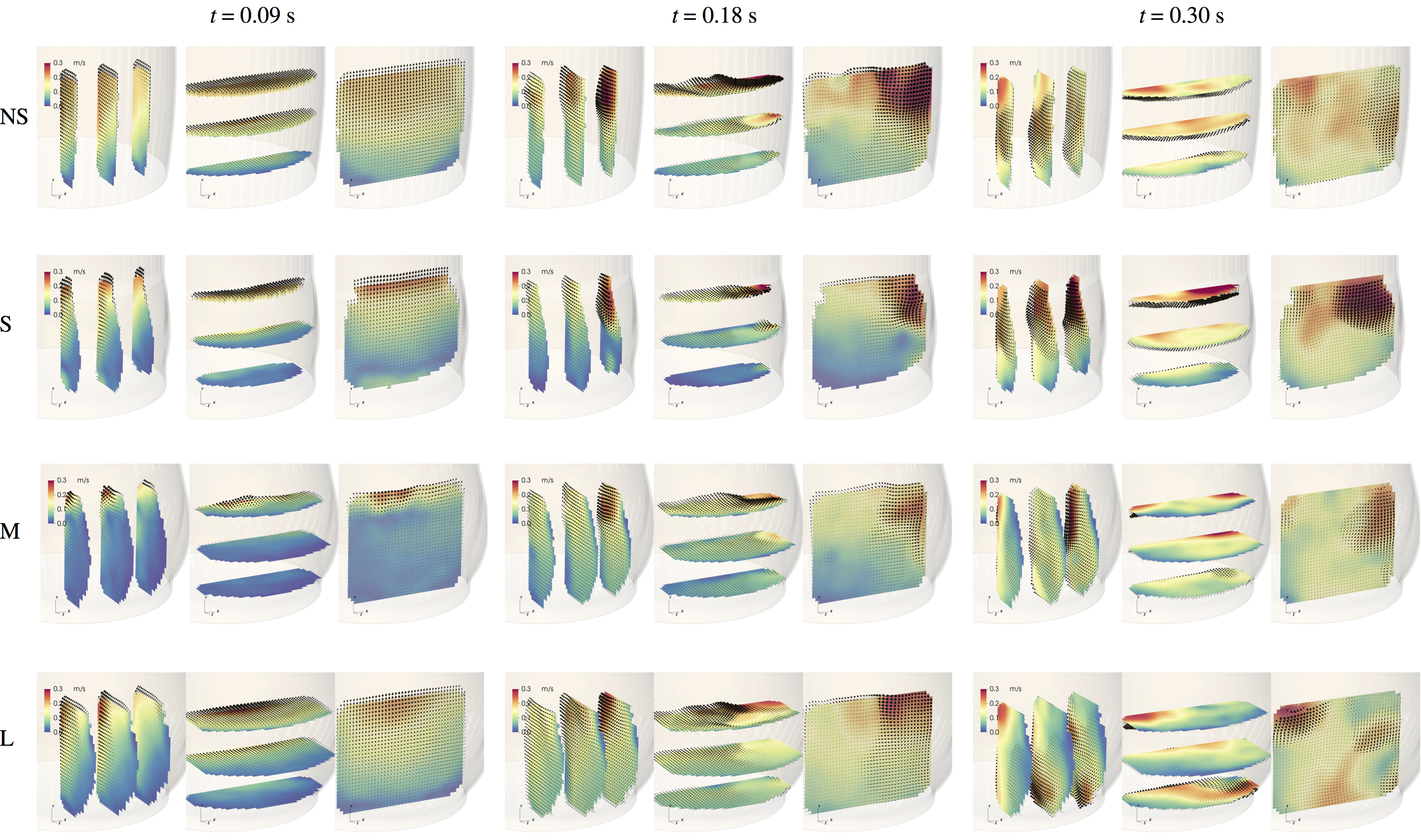

Supplement: S2 Fig — Sinus flow mean velocity magnitude in the non-sinus (NS), the small (S), the medium (M), and the large (L) aortic root configuration, during valve opening (t = 0.09), mid-systole (t = 0.18), and valve closure (t = 0.30). (PNG) [file pone.0194384.s003.png]
